# LncRNA DNM3OS promotes proliferation and inhibits apoptosis through modulating IGF1 expression by sponging MiR-126 in CHON-001 cells

**DOI:** 10.1186/s13000-019-0877-2

**Published:** 2019-09-16

**Authors:** Di Ai, Fang Yu

**Affiliations:** Department of Joint Surgery, Beijing Shijitan Hospital, CMU, No. 10 Tieyi Road, Yangfangdian, Haidian District, Beijing, 100038 China

**Keywords:** LncRNA dynamin 3 opposite strand, MiR-126, Type insulin-like growth factor-1, Osteoarthritis, Proliferation

## Abstract

**Background:**

As a degenerative disease, osteoarthritis (OA) greatly affects aged population. The human chondrocyte cell line CHON-001, derived from normal human articular cartilage, has been widely used in vitro in osteoarthritis models. In order to better understand the underlying mechanism of OA pathogenesis, this study was conducted to explore the effects of LncRNA dynamin 3 opposite strand (DNM3OS) on CHON-001 cells.

**Methods:**

The expression levels of and correlation between DNM3OS and miR-126 that derived from OA and non-OA tissues were determined by quantitative real time (qRT)-PCR and Spearman’s correlation analysis. Cell viability, clone, migration, invasion and apoptosis were respectively determined by cell counting kit-8, colony formation, wound healing assay, transwell and flow cytometry. The target genes were predicted by starbase V2 and targetscan 7.2 and confirmed by luciferase reporter assay. The expressions of apoptosis-related factors were detected by Western blot.

**Results:**

The expression of DNM3OS was down-regulated in OA patients. Functional assays demonstrated that ectopic expression of DNM3OS promoted the proliferation and inhibited apoptosis of CHON-001 cells, and that knocking down DNM3OS suppressed cell proliferation and induced apoptosis. Mechanistic investigation revealed that DNM3OS physically bound to the promoter of miR-126 and suppressed miR-126 expression. Decreased expression of DNM3OS was negatively correlated with miR-126 in OA patients. Furthermore, the effects of siDNM3OS on inhibiting cell proliferation and promoting apoptosis were partially reversed by miR-126 inhibitor. Meanwhile, type insulin-like growth factor-1 (IGF1) was identified as a target gene for miR-126 and was negatively associated with the miR-126 expression. Overexpressed IGF1 restored the effects of miR-126 mimic in suppressing cell proliferation and promoting apoptosis.

**Conclusion:**

Our results showed that DNM3OS could affect the CHON-001 cell proliferation and apoptosis by regulating IGF1 by sponging miR-126.

## Background

Osteoarthritis (OA) is a common degenerative disease characterized by the reduction of articular chondrocytes and destruction of joint matrix. Patients with severe OA suffer from joint deformity and loss of joint function [1, 2]. Epidemiological statistical results showed that the prevalence of OA in patients aged over 40 years old in China was about 30, and 60%–70% among patients aged above 65 years [3, 4]. With the aging of Chinese population, the occurrence of OA is increasing and becomes a serious social and public health problem. Due to the complex pathological process of OA and the mechanisms involved, different strategies such as chondrocyte transplantation and bone marrow mesenchymal stem cell transplantation and matrix-induced autologous chondrocyte transplantation have been clinically applied for treating different pathological processes in relation to the mechanisms of OA, though not satisfactory, these options still achieved a certain effect [5–8]. Therefore, further research on the pathological mechanism of OA and discovering more effective prevention targets are still two difficult problems that need to be solved.

Considering that OA has a typical heritability, gene therapy on OA has attracted much attention. As the expression of the gene in vivo is difficult to be controlled, and the selection and safety of the vector requires to be further verified, therefore, the OA therapy is still in the stage of animal experiment and in vitro research [9, 10]. Non-coding RNA was considered as a non-functional RNA, and its importance has gradually been recognized as research advanced [11, 12]. As a subclass of non-coding RNA, long-chain non-coding RNA is more than 200 nt in length and has stronger tissue specificity and cell specificity than coding RNA. Long-chain non-coding RNA has different expressions in different cells, tissues and developmental stages and is related to the occurrence and development of various diseases [13–15]. Dynamin 3 opposite strand (DNM3OS), which is a 7.9-kb antisense transcript, is located within the 14th intron of the mouse Dnm3 gene [16]. The DNM3OS knock-out mice constructed by Watanabe Laboratories found that mice with DNM3OS knockout showed skeletal dysplasia and was accompanied by abnormal fat development [17]. A recent study proved that DNM3OS promoter containing regulatory elements are associated to chondrogenesis [18]. It is also demonstrated that enriched lncRNAs in the cytoplasm typically participates in post-transcriptional regulation by interacting with miRNAs or mRNAs [19, 20]. MiR-126 inhibitor confirmed IL-1β-induced chondrocyte apoptosis and inflammatory response [20]. In combination with published experiments, we speculated that targeting DNM3OS is possibly a promising therapeutic approach for OA.

However, DNM3OS expression in OA and its role in OA development remain unclear. Thus, this study aims to investigate the roles of DNM3OS in the development of OA and the potential molecular mechanisms involved. This study provides a theoretical basis for OA treatment and novel perspective for clinical therapy.

## Methods

### OA tissues

The OA cartilage was isolated from the knees of 56 patients undergoing total knee arthroplasty in Beijing Shijitan Hospital, CMU, while normal articular cartilages were obtained from 42 normal persons without OA, and all patients signed informed consent. The study was approved by the Human Ethics Committee of Beijing Shijitan Hospital, CMU. Patients were enrolled if they met the following inclusion criteria: X-ray showed osteophyte formation at the edge of the joint, the joint fluid examination was consistent with osteoarthritis (clear and transparent, sticky, WBC < 2 × 10^9^/L), patients aged 45–75 years old and have not taken the drug for 7 days. The tissues were collected and stored at − 80 °C.

### Cell culture

Human chondrocyte cell line CHON-001 cells were obtained from American Type Culture Collection (ATCC; Manassas, Virginia, USA). The cells were grown in cultured in Dulbecco’s Modified Eagle’s Medium (DMEM, Gibco, Grand Island, New York) containing 10% fetal bovine serum (FBS; Gibco) and 0.1 mg/ml G-418 (Gibco). The cells were cultured in 5% CO_2_ at 37 °C in an incubator.

### Transfection

The small interfering DNM3OS (siDNM3OS) and the negative control (siNC) were acquired from Shanghai GenePharma Co., Ltd. (Shanghai, China). The siRNA sequences were as follows: siDNM3OS sense: 5′-CAACTAGTGTTCAACTATA-3′ and antisense: 5′-GATTGATAATTCAAGTGTT-3′; siNC sense: 5′-TGCCTTGGAGGCCACATAAA-3′ and antisense: 5′-ATCATGCCCCAAACCCATTA-3′. Lipofectamine^TM^ 3000 (Thermo Fisher Scientific, Inc.) was performed following the manufacturer’s instructions. In brief, 2 μl Lip3000, 40 pmol of small interfering RNA and siNC (GenePharma) were respectively mixed in 50 μl serum-free medium and incubated at room temperature for 15 min. The lipid compounds were diluted in 300 μl serum-free medium and 600 μl medium containing FBS to produce 1 ml mixture and then incubated with the cells at 37 °C with 5% CO_2_ for subsequent experiments. For the expression vectors used to up-regulate the target gene, 5 μl Lip3000 and 2 μg DNA (GenePharma) were mixed in 125 μl DMEM and incubated at room temperature for 5 min. 4 μl Lip3000 and 125 μl DMEM were mixed and incubated at room temperature for 5 min. Then, the two mixtures were mixed to form a new mixture. After 5 min, the mixture was used to treat the cells at 37 °C with 5% CO_2_ for subsequent experiments.

### Cell counting kit 8 (CCK-8)

CHON-001 cells viability was detected by CCK-8, and the cells were plated on a 96-well plate at a density of 1 × 10^5^ cells/well. After culturing the transfected cells for 24, 48 and 72 h, 10 μl CCK8 reagent was added to the hole and incubated at 37 °C with 5% CO_2_ for 2 h. A microplate reader (Model 550, Bio-Rad Laboratories, Inc., Hercules, CA, USA) was used to determine the OD at an absorbance of 450 nm of each well in different cell groups.

### Cloning experiment

48 h after the transfection, the cells were treated by 0.3% soft agar and incubated in 6-well plates at 500 cells/well in DMEM containing 10% FBS at 37 °C. The medium was replaced by unsalted DMEM every 2 days. After 14 days, the medium was drained and washed by PBS for 2–3 times. 2 ml 4% paraformaldehyde was added to each well and allowed to stand for 20 min. Paraformaldehyde was removed, 1 ml Giemsa staining solution (solution A: B solution = 1:1.25) was added and held for 20 min, and then the staining solution was washed off. The 6-well plate was slowly rinsed in running water and dried at room temperature. The number of clones more than 10 cells was observed under a microscope (Olympus, Tokyo, Japan). Plate clone formation rate = number of clones formed/number of cells inoculated × 100%.

### Wound healing assay

After transfecting CHON-001 cells for 48 h, a straight gap was created by a 200 μl sterile tip in the middle of the well. The cells were washed by DMEM twice for smoothing the edges of the scratch and removing floating cells. After incubating the cells at 37 °C in 5% CO_2_ for 0 and 48 h, the migration images of the cells were observed under a microscope (Keyence, Osaka, Japan), the distance of cell migration was visualized and images were taken using image-Pro Plus Analysis software (version 5.0; Media Cybernetics Company, USA).

### Transwell

8 μm transwell chamber (3413, American Corning Company, New York, USA) was placed on a 24-well plate coated with a layer of 50 μl matrigel (BD, Biosciences). Materigel was diluted at 1:8, and coated on the upper chamber of the bottom membrane of the Transwell chamber and placed in an incubator at 37 °C for 30 min to polymerize Materigel into gel. 200 μl cell suspension containing 1 × 10^5^ cells were added to the upper layer of the transwell chamber, while 600 μl 20% FBS were added to the lower chamber. After incubating the 24-well plate in an incubator for 24 h, the un-invaded cells were gently wiped off using a cotton swab, and the chamber was air-dried appropriately. The cells were fixed in 4% paraformaldehyde for 15 min and stained with 0.1% crystal violet for 20 min. The cells from 5 random fields were observed and counted under a microscope.

### Flow cytometry

Apoptosis detection kit (BD Biosciences Medical Devices Shanghai Co., Ltd., Shanghai, China) was used to detect CHON-001 cells apoptosis. The cells (5 × 10^5^ cells/well) were seeded in 6-well plates, to reach 85% cell density. 48 h after the transfection, the cells were digested, centrifuged and washed twice by PBS. The cells were added with 100 μl of 1 × Annexin-V Binding Buffer, 5 μl FITC-labeled Annexin-V (20 μg/ml) and 5 μl of PI (50 μg/ml) and kept at room temperature for 20 min. Flow cytometer (version 10.0, FlowJo, FACS CaliburTM, BD, Franklin Lakes, NJ, USA) was used to determine the cells apoptosis.

### Bioinformatics prediction

The target genes of DNM3OS was predicted by bioinformatics prediction system (StarBase v2.0) (http://starbase.sysu.edu.cn/index.php), and the potential target genes for miR-126 were predicted by targetscan 7.2 online software (http://www.targetscan.org/vert_72/) according to the manufacturer’s instruction.

### Dual luciferase reporter assay

Dual luciferase reporter gene analysis was performed to confirm the direct binding abilities of LncRNA-DNM3OS/microRNA-126 and microRNA-126/IGF1. After 48 h of transfection, luciferase reporter assay system (Promega Corporation) was performed to measure the luciferase activity in Lmax II luminescence meter (Molecular Devices, LLC, Sunnyvale, CA, USA). Luciferase activity was defined as the standardization of firefly luciferase activity.

### Reverse transcription quantitative polymerase chain reaction

OA tissues and transfected CHON-001 cells were incubated in an incubator for 24 h, and total RNA was extracted using TRIzol® reagent (Thermo Fisher Scientific, Inc.). A NanoDrop™ 2000 spectrophotometer (Thermo Fisher Scientific, Inc.) was conducted to determine RNA quality (an A260/A280 ratio between 1.8 and 2.0 was required for generating cDNA). The oligo-dT or stem-loop reverse transcriptase primers (Takara Bio, Inc., Otsu, Japan) were used to obtain cDNA and the reaction conditions were as follows: at 42 °C for 60 min, at 70 °C for 5 min and preserved at 4 °C. QPCR was performed using the SYBR® Premix Ex Taq™ II (Takara Bio Inc.) on real-time PCR Detection System (ABI 7500, Life Technology, United States). PCR reaction conditions were as follows: pretreatment at 95 °C for 10 min, followed by 40 cycles at 94 °C for 15 s, at 60 °C for 1 min, finally at 60 °C for 1 min and preserved at 4 °C. 2^−ΔΔCq^ method was used to process the data. The primers used in this experiment were listed as Table 1.

**Table 1:** Primers for RT-qPCR

Genes	Forward sequence (5′–3′)	Reverse sequence (5′–3′)
DNM3OS	GTCAGCGCAGCAGAATTCAG	CGGCAGTCTTTTCTCAGCAG
IGF1	CATGCCT GCTCAGAAGGGTA	GCCTCTGATCCTTGAGGTGA
miRNA-126	GGGTCGTACCGTGAGTAAT	CAGTGCGTGTCGTGGAGT
U6	CTCGCTTCGGCAGCACA	TGGTGTCGTGGAGTCG

### Western blot

CHON-001 cells were treated by plasmids, siRNAs and the corresponding control and then cultured in an incubator for 48 h. Total proteins were collected by Radio-Immunoprecipitation Assay (RIPA, Beyotime, China). BCA Protein Assay Kit (Pierce) was used to measure the concentrations of proteins, which were adjusted to a concentration of 6 μg/μl using 1 × loading and DEPC water. 5 μl of the samples was separated on 10% SDS-PAGE gels and then transferred to polyvinylidene fluoride membrane (PVDF, Millipore, and USA). After blocking the membrane in 5% non-fat milk in PBST (0.1% Tween 20 in phosphate-buffered saline (PBS)) for 1 h, the membrane was probed by primary antibody overnight at 4 °C, washed by PBST 3 times and then incubated with secondary antibody (horseradish peroxidase (HRP)-conjugated goat/bovine anti-mouse/rabbit/goat IgG, 1:2000; sc-516,102/sc-2357/sc-2350; Santa Cruz Biotechnology, Inc. Dallas, TX, USA) at room temperature. After 2 h, the membrane was washed by PBST for 3 times. A developer (EZ-ECL kit; Biological Industries BI) was used for development, and the gray value of the strips were determined and counted by ImageJ (version 5.0; Bio-Rad, Hercules, CA, USA). The antibodies used were anti-GAPDH (36 kDa; mouse; 1:2000; ab8245; Abcam), anti-Bax (21 kDa; rabbit; 1:1000; ab32503; Abcam), anti-Bcl-2 (26 kDa; rabbit; 1:1000; ab59348; Abcam), anti-cleaved caspase-3 (17 kDa; rabbit; 1:1000; ab2302; Abcam) and anti-IGF1 (22 kDa; Goat; 1:1000; ab106836; Abcam).

### Statistical analysis

Data were shown as the mean ± SD. Statistical software Prism 7 (GraphPad Software, Inc., San Diego, CA, USA) was used for statistical analysis. Comparison between two groups was conducted by Student’s t-test. The negative correlation between DNM3OS and miR-126 expression levels in OA and normal samples was analyzed by Spearman’s correlation analysis. *P* < 0.05 was considered as statistically significant.

## Results

### DNM3OS was low-expressed in OA tissues, had negative correlation with miR-126 and promoted CHON-001 cells viability

**Fig. 1:**
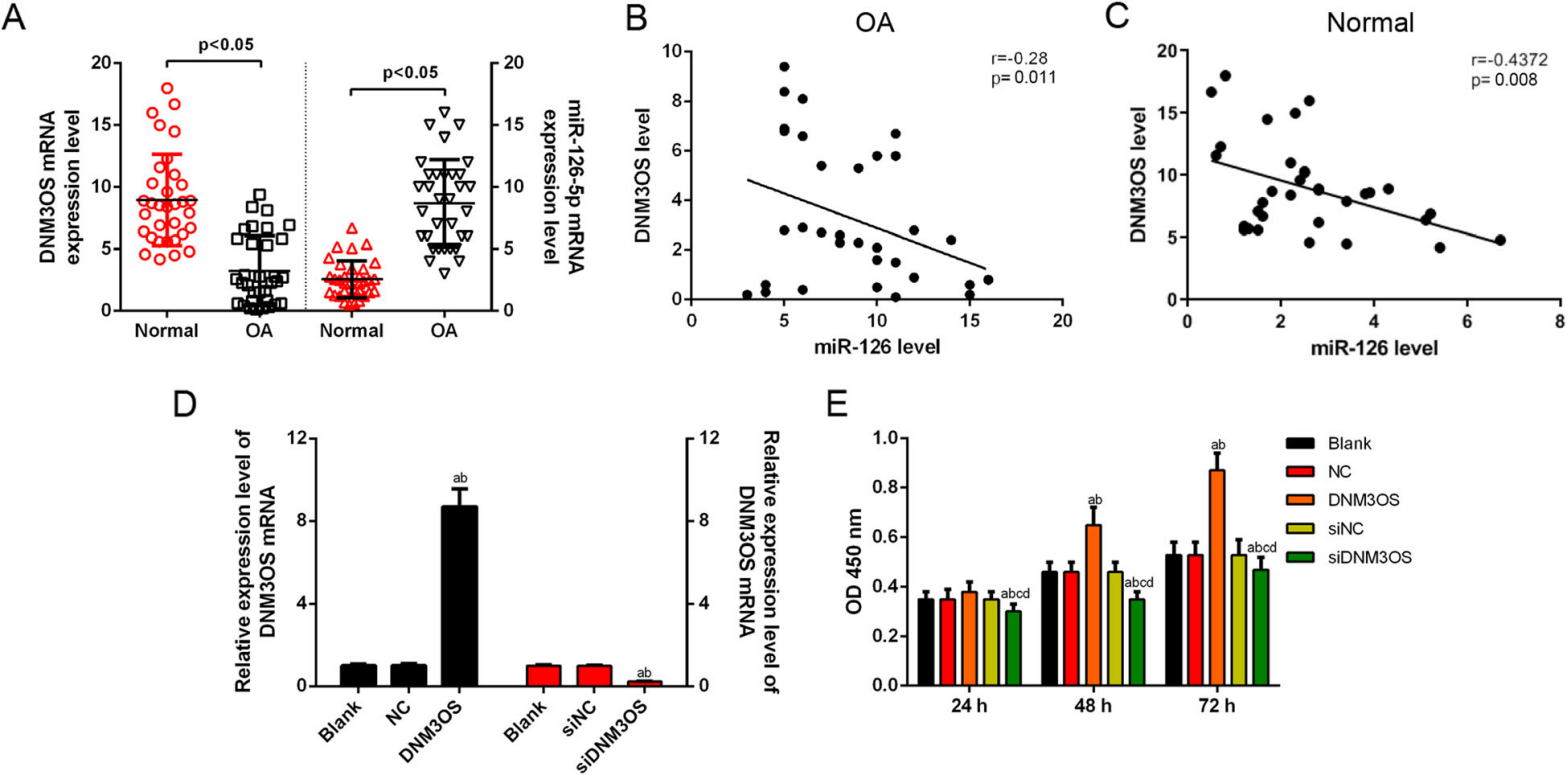
DNM3OS was low-expressed in OA tissues, had negative correlation with miR-126 and promoted CHON-001 cells viability. (**a**) The expression of lncRNA DNM3OS in human OA tissues was significantly lower, while miR-126 was obviously higher than in normal tissues using RT-qPCR. (**b**) The expression of DNM3OS was negatively correlated with the miR-126 level in OA samples by Spearman’s correlation analysis. (**c**) The expression of DNM3OS was negatively correlated with the miR-126 level in normal samples by Spearman’s correlation analysis. (**d**) The transfection efficiency of DNM3OS was confirmed by RT-qPCR. (**e**) CHON-001 cells viability was improved or inhibited by DNM3OS or silencing DNM3OS by CCK-8 assay. ^a^*P* < 0.05 vs. Blank, ^b^*P* < 0.05 vs. NC, ^c^*P* < 0.05 vs. DNM3OS, ^d^*P* < 0.05 vs. siNC. siDNM3OS: small interfering dynamin 3 opposite strand; NC: negative control; OD: optical density

To explore whether DNM3OS and miR-126 changed in OA chondrocytes, the expression levels of DNM3OS and miR-126 were detected by qRT-PCR assay in 45 OA patients and 20 normal patients. The data showed that the DNM3OS expression was significantly down-regulated, while miR-126 was up-regulated in the OA group, compared with the normal group (Fig. 1a). Furthermore, in the OA patient samples, Spearman’s correlation analysis (Fig. 1b, c) found that the level of DNM3OS was negatively correlated with miR-126 expression. To investigate the functions of DNM3OS in OA, the expression level of DNM3OS was successfully increased or decreased by DNM3OS overexpression vector or small interfering DNM3OS (siDNM3OS) in CHON-001 cells, compared with the counterpart negative control (Fig. 1d). CCK-8 assay findings showed that the up-regulation of DNM3OS improved CHON-001 cells viability at 48 h and 72 h, while siDNM3OS inhibited cell viability at 24, 48 and 72 h (Fig. 1e).

### Over-expression of DNM3OS enhanced proliferation and suppressed apoptosis in CHON-001 cells, while the inhibition of DNM3OS showed opposite effects

**Fig. 2:**
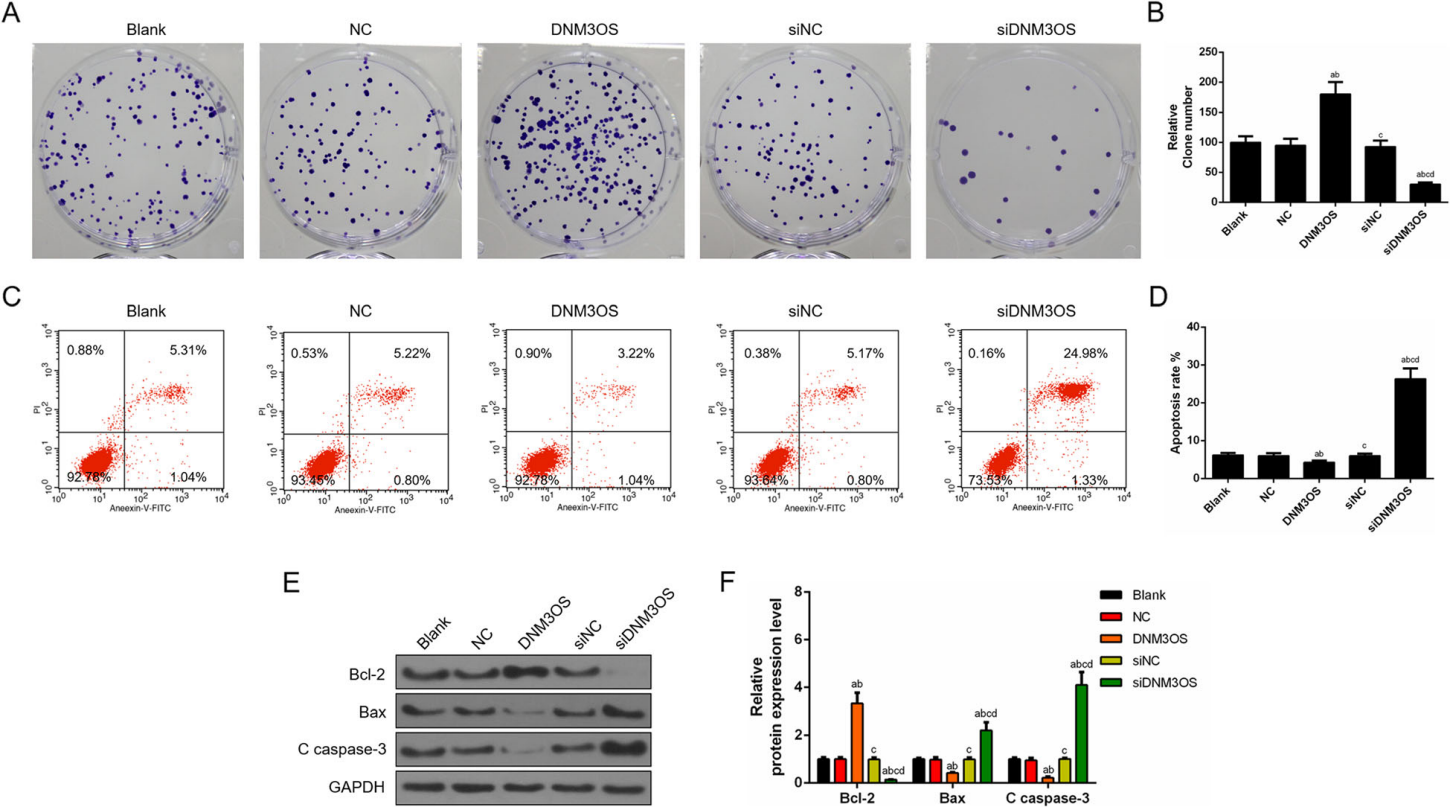
Over-expression of DNM3OS enhanced proliferation and suppressed apoptosis in CHON-001 cells, while DNM3OS silencing had opposite effects. (**a**) CHON-001 cells proliferation was improved by DNM3OS, while silencing DNM3OS inhibited CHON-001 cells proliferation as indicated by cloning formation experiment. (**b**) The number of proliferated cells was quantified. (**c**) CHON-001 cells apoptosis was inhibited by DNM3OS, while DNM3OS silencing induced apoptosis as indicated by flow cytometry. (**d**) The proportion of apoptosis was quantified. (**e**) The apoptosis-related proteins (Bcl-2, Bax and c caspase 3) were detected by Western blot. (**f**) The relative levels of proteins described in (**e**) were counted by GAPDH as normalization. ^a^*P* < 0.05 vs. Blank, ^b^*P* < 0.05 vs. NC, ^c^*P* < 0.05 vs. DNM3OS, ^d^*P* < 0.05 vs. siNC. siDNM3OS: small interfering dynamin 3 opposite strand; NC: negative control; Bcl-2: B-cell lymphoma-2; Bax: Bcl2-associated X protein

We identified that cell viability was increased by overexpressed DNM3OS. Next, the cell proliferation was determined by cloning formation experiment, and the data revealed that the up-regulation of DNM3OS greatly accelerated cell proliferation, while the knockdown of DNM3OS significantly inhibited the cell proliferation (Fig. 2a, b). Moreover, the effect of DNM3OS on the apoptosis of chondrocytes CHON-001 cells was assessed by flow cytometry, and the apoptosis results showed that the up-regulation of DNM3OS significantly inhibited cells apoptosis, while the down-regulation of DNM3OS obviously triggered cells apoptosis (Fig. 2c, d). Consistently, western blot assay showed that DNM3OS overexpression led to the suppression of apoptosis and knocking down DNM3OS promoted cell apoptosis, which were supported by the expression changes of apoptosis-related proteins Bax, Bcl2 and cleave caspase-3 (Fig. 2e, f).

### CHON-001 cells migration and invasion were facilitated by up-regulation of DNM3OS, while the knockdown of DNM3OS exhibited the opposite results

**Fig. 3:**
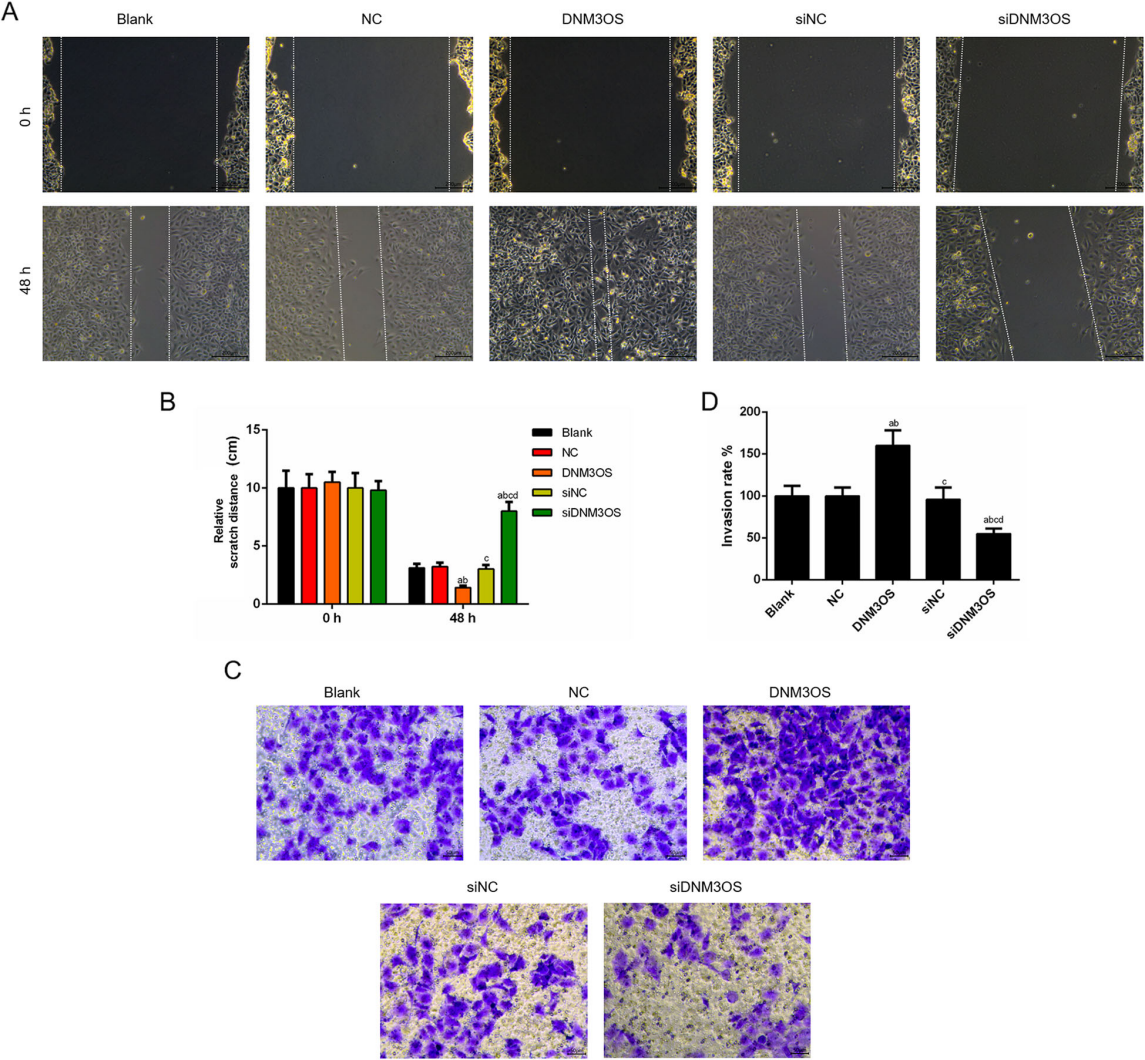
CHON-001 cell migration and invasion were facilitated by the up-regulation DNM3OS, while the knockdown of DNM3OS showed opposite results. (**a**) CHON-001 cells migration was promoted by DNM3OS, while silencing DNM3OS inhibited cells migration as indicated by wound healing assay. (**b**) The distance of scratch was quantified. (**c**) CHON-001 cells invasion was enhanced by DNM3OS, while silencing DNM3OS suppressed cells invasion as indicated by transwell assay. (**d**) The number of invasion cells was quantified. ^a^*P* < 0.05 vs. Blank, ^b^*P* < 0.05 vs. NC, ^c^*P* < 0.05 vs. DNM3OS, ^d^*P* < 0.05 vs. siNC. siDNM3OS: small interfering dynamin 3 opposite strand; NC: negative control

Wound-healing assay and Transwell assay were performed to further investigate the biological effects of DNM3OS on the migration and invasion of CHON-001 cells. As shown in Fig. 3a and b, the distance of scratch was shorter in DNM3OS group than that in NC group, and the scratch distance in siDNM3OS group increased significantly compared with that in siNC group (Fig. 3a, b). Cell invasion was increased by the treatment of overexpressed DNM3OS, and ability of cell invasion induced by the knockdown of DNM3OS under the treatment of DNM3OS siRNA was weaker than siRNA scramble treatment (Fig. 3c, d).

### LncRNA DNM3OS acted as a molecular sponge of miR-126

**Fig. 4:**
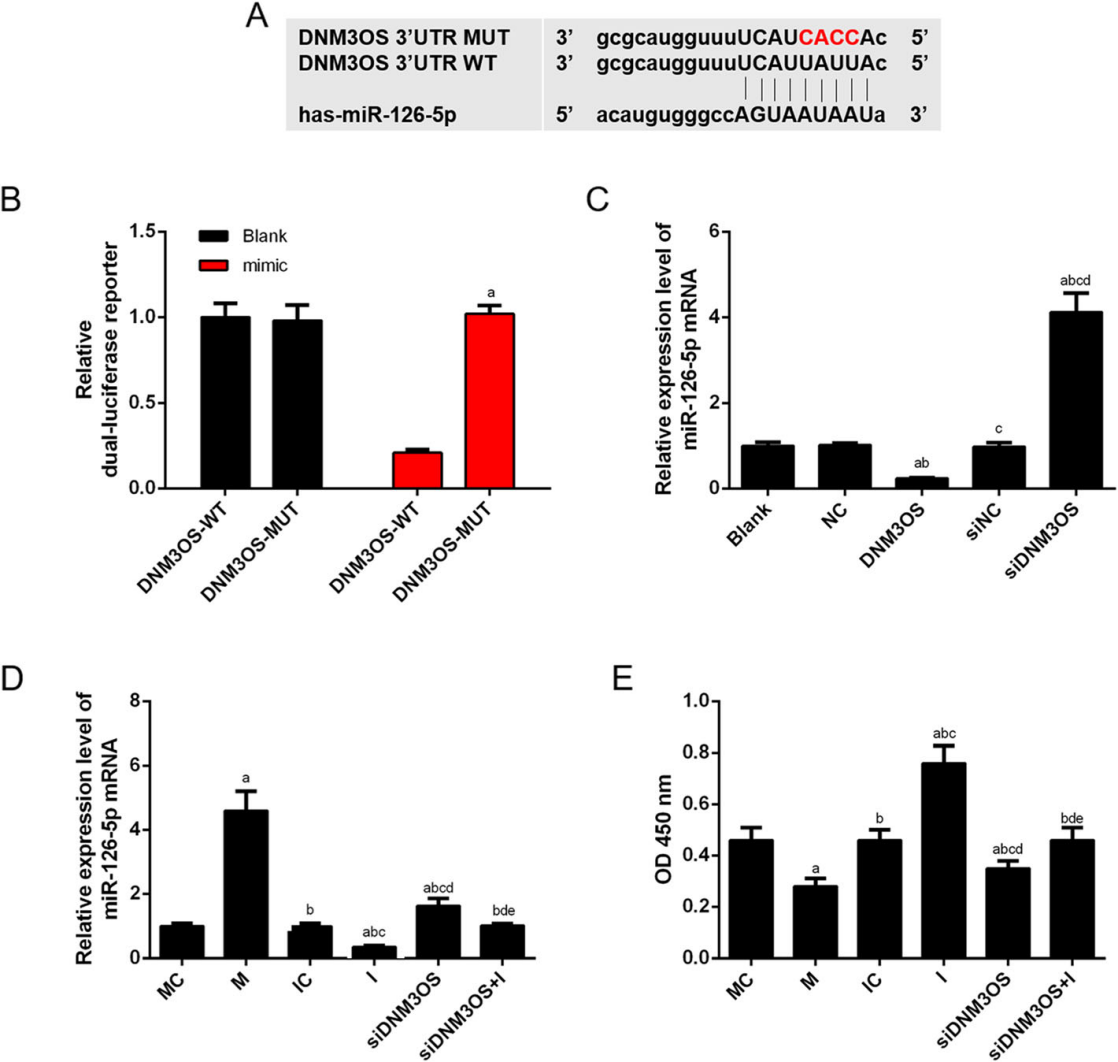
LncRNA DNM3OS acted as a molecular sponge of miR-126. (**a**) The predicted binding sequences of miR-126 in DNM3OS using starBase v2.0. (**b**) The luciferase intensity of cells transfected with DNM3OS wild-type and miR-126 was significantly reduced. (**c**) The expression of miR-126-5p was negatively associated to DNM3OS in CHON-001 cells by RT-qPCR. (**d**) MiR-126-5p inhibitor reversed the promotion effects of siDNM3OS on miR-126-5p level as indicated by RT-qPCR. (**e**) The inhibitory effects of siDNM3OS on cells viability were weakened by miR-126 inhibitor. ^a^*P* < 0.05 vs. DNM3OS-WT or Blank or MC, ^b^*P* < 0.05 vs. NC or M, ^c^*P* < 0.05 vs. DNM3OS or IC, ^d^*P* < 0.05 vs. I, ^e^*P* < 0.05 vs. siDNM3OS. WT: wild-type; MUT: mutated-type; NC: negative control; mimic: miR-126 mimic; M: miR-126 mimic; I: miR-126 inhibitor; MC: miR-126 mimic control; IC: miR-126 inhibitor control; siDNM3OS: small interfering dynamin 3 opposite strand

To investigate the underlying mechanism of DNM3OS in OA, online software starBase v2.0 was used to observe the miRNAs interacting with DNM3OS. Interestingly, we found that some complementary bases of miR-126-5p had the sequences of DNM3OS (Fig. 4a), and the luciferase reporters results further confirmed that the overexpression of miR-126 was suppressed by the binding RNA DNM3OS-Wild-type (WT) construct, while RNA DNM3OS-Mutated-type (MUT) did not change significantly (Fig. 4b). Moreover, miR-126 expression was greatly suppressed by overexpressed DNM3OS but enhanced by DNM3OS silencing compared with their corresponding controls (Fig. 4c). In addition, we investigated whether miR-126 was involved in the regulation of lncRNA DNM3OS on CHON-001 cell viability. The CHON-001 cells were successfully transfected with miR-126 mimic, inhibitor, si-DNM3OS and corresponding controls (Fig. 4d), and we found that the cell viability was decreased or increased by mimic or inhibitor, moreover, co-transfecting the cells with si-DNM3OS and miR-126 inhibitor promoted cell viability (Fig. 4e).

### Down-regulation of miR-126 abolished the DNM3OS knockdown-mediated inhibition of cell proliferation and promotion of cell apoptosis

**Fig. 5:**
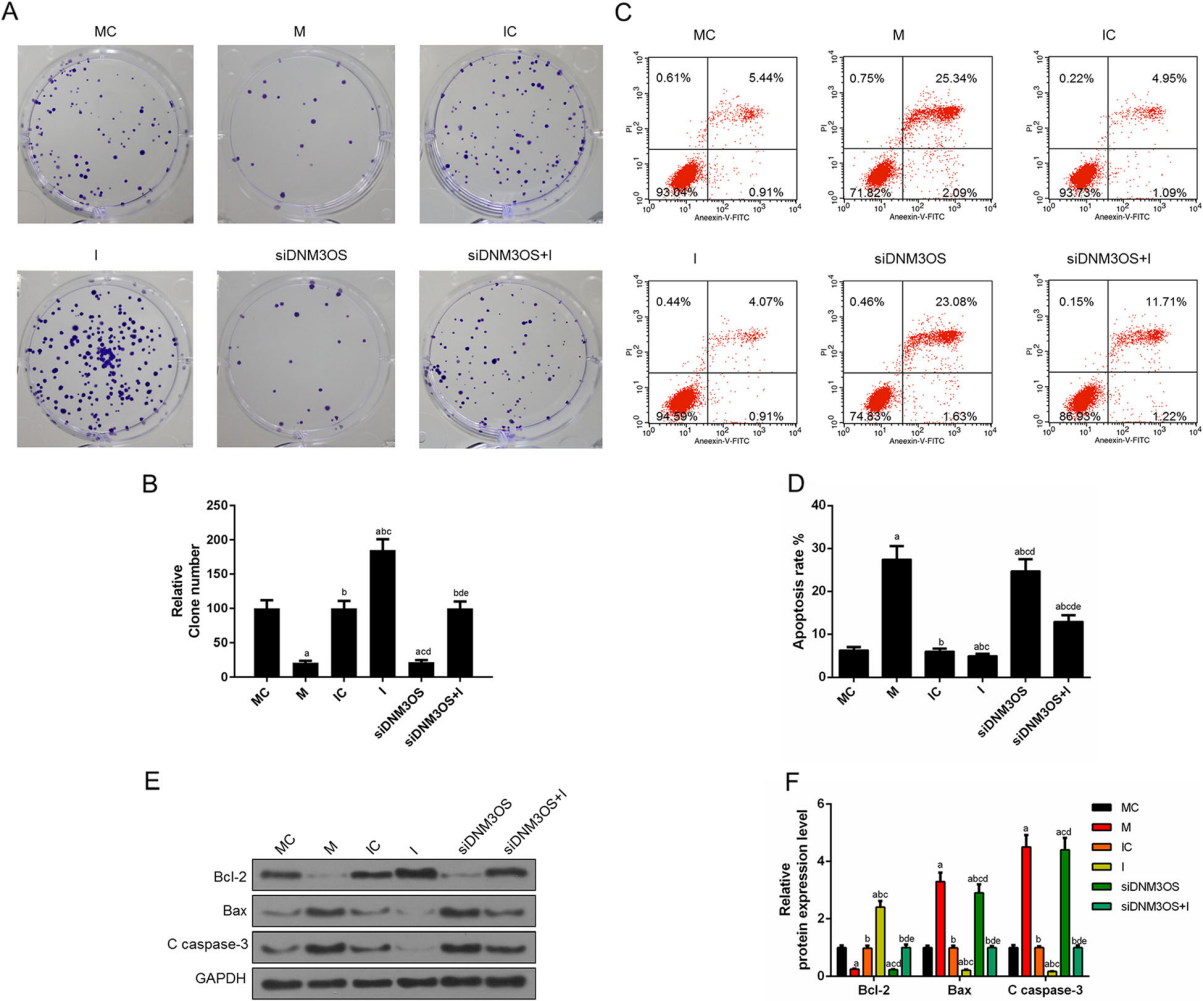
The down-regulation of miR-126 abolished the DNM3OS knockdown-mediated inhibition of cells proliferation and promotion of cell apoptosis. (**a**) Co-transfecting si-DNM3OS and miR-126 inhibitor promoted cell proliferation as shown by cloning formation experiment. (**b**) The number of proliferated cells was quantified. (**c**) Cells apoptosis was triggered by silencing DNM3OS, which partially reversed by miR-126 inhibitor. (**d**) The proportion of apoptosis was quantified. (**e**) The apoptosis-related proteins (Bcl-2, Bax and c caspase 3) were detected by Western blot. (**f**) The relative levels of proteins described in (**e**) were counted by GAPDH as normalization. ^a^*P* < 0.05 vs. MC, ^b^*P* < 0.05 vs. M, ^c^*P* < 0.05 vs. IC, ^d^*P* < 0.05 vs. I, ^e^*P* < 0.05 vs. siDNM3OS. M: miR-126 mimic; I: miR-126 inhibitor; MC: miR-126 mimic control; IC: miR-126 inhibitor control; siDNM3OS: small interfering dynamin 3 opposite strand; Bcl-2: B-cell lymphoma-2; Bax: Bcl2-associated X protein

Rescue experiments were conducted to determine whether DNM3OS was dependent on the regulation of miR-126 in producing biological effects. We infected CHON-001 cells with miR-126 mimic, inhibitor and siDNM3OS to determine cell proliferation and apoptosis using corresponding biological methods. The data showed that the cell proliferation was inhibited by miR-126 mimic but increased by inhibitor. Cell proliferation was promoted by the co-transfection with si-DNM3OS and miR-126 inhibitor (Fig. 5a, b). The flow cytometry results showed that miR-126 mimic significantly induced cells apoptosis, while the inhibitor obviously suppressed cells apoptosis. Furthermore, cell apoptosis was triggered by silencing DNM3OS but partially reversed by miR-126 inhibitor (Fig. 5c, d). Concomitantly, the expression changes of apoptosis-related proteins Bax, Bcl2 and cleave caspase-3 were observed using Western blot, and the results showed that miR-126 mimic promoted cells apoptosis, which was inhibited by the inhibitor, moreover, DNM3OS knockdown promoted apoptosis, which was observed inhibited by miR-126 inhibitor (Fig. 5e, f).

### Down-regulation of DNM3OS suppressed the migration and invasion by acting as a miR-126 sponge

**Fig. 6:**
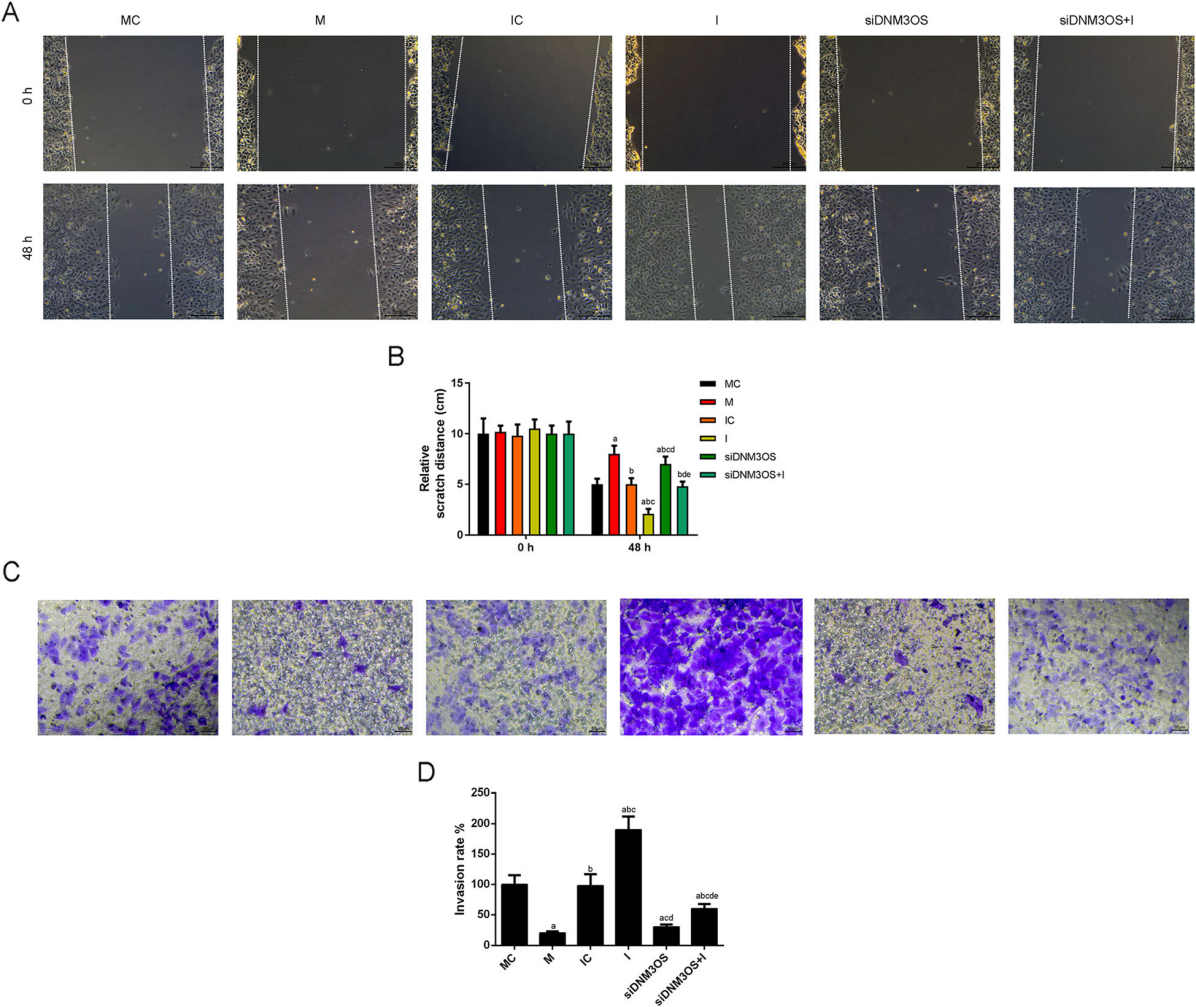
DNM3OS down-regulation repressed migration and invasion by acting as a miR-126 sponge. (**a**) CHON-001 cells migration was inhibited by silencing DNM3OS, which reversed by miR-126 inhibitor using wound healing assay. (**b**) The distance of scratch was quantified. (**c**) CHON-001 cells invasion was inhibited by silencing DNM3OS, which was reversed by miR-126 inhibitor using transwell assay. (**d**) The number of invasion cells was quantified. ^a^*P* < 0.05 vs. MC, ^b^*P* < 0.05 vs. M, ^c^*P* < 0.05 vs. IC, ^d^*P* < 0.05 vs. I, ^e^*P* < 0.05 vs. siDNM3OS. M: miR-126 mimic; I: miR-126 inhibitor; MC: miR-126 mimic control; IC: miR-126 inhibitor control; siDNM3OS: small interfering dynamin 3 opposite strand

To further investigate the regulatory relation between DNM3OS and miR-126, the CHON-001 cells migration and invasion were measured by wound healing assay and transwell, respectively. The results revealed that the distance of scratch was increased in miR-126 mimic but decreased in inhibitor-treated cells, while the distance of scratch was decreased in DNM3OS-inhibitor cells compared with control cells (Fig. 6a, b). Moreover, the invasion ability was down-regulated in miR-126 mimic but up-regulated in inhibitor groups in comparison to the control group, while inhibitor reversed the inhibitory effect of silencing DNM3OS on cells invasion (Fig. 6c, d).

### MiR-126 directly targets IGF1 in CHON-001 cells

**Fig. 7:**
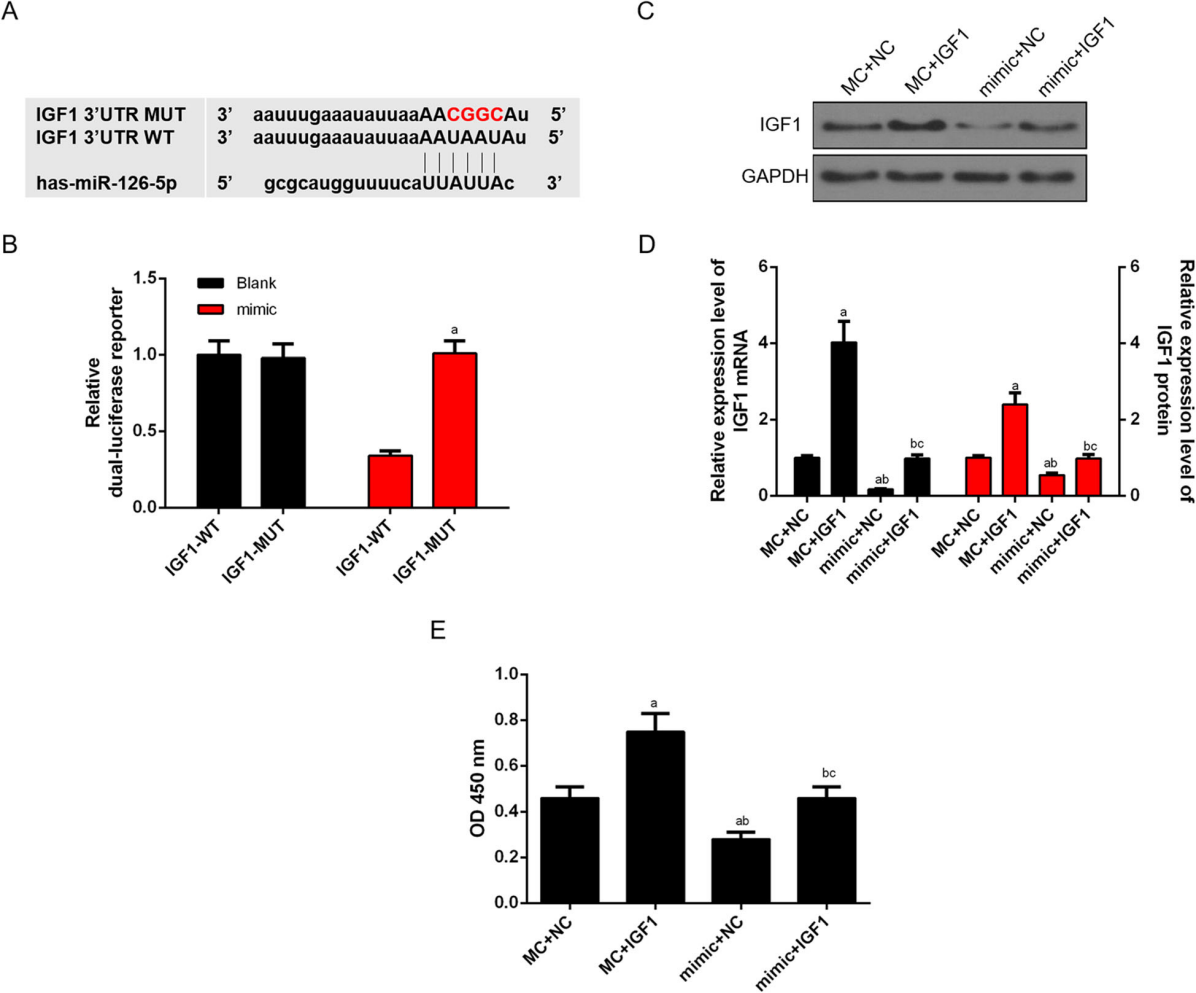
MiR-126 directly targeted IGF1 in CHON-001 cells. (**a**) The predicted binding sequences of IGF1 in miR-126 using targetscan 7.2. (**b**) The luciferase intensity of cells transfected with miR-126 wild-type and IGF1 was significantly reduced. (**c**) The protein level of IGF1 was decreased in miR-126 mimic-treated cells. (**d**) The mRNA and protein level of IGF1 were quantified. (**e**) CHON-001 cells viability was higher in mimic+IGF1 than that in mimic group. ^a^*P* < 0.05 vs. IGF1-WT or MC + NC, ^b^*P* < 0.05 vs. MC + IGF1, ^c^*P* < 0.05 vs. mimic+NC. Mimic: miR-126 mimic; MC: miR-126 mimic control; WT: wild-type; MUT: mutated-type

To determine the miR-126 target genes involved in OA progression, targetscan 7.2 was used to predict the target gene of miR-126. Interestingly, one highly conserved putative binding site was observed in the 3′-UTR of IGF1 (Fig. 7a). Furthermore, the luciferase reporter results showed that the binding RNA IGF1-WT construct suppressed overexpressed miR-126, while RNA IGF1-MUT did not change significantly (Fig. 7b). Additionally, RT-qPCR and Western blot analysis revealed that the restoration of miR-126 expression suppressed the IGF1 mRNA and protein, which were enhanced by the up-regulation of IGF1 (Fig. 7c, d). CCK-8 assay experiment found that cell viability was increased by IGF1 but decreased by miR-126 mimic. Co-transfecting IGF1 and miR-126 mimic, the cell viability was higher in IGF1-miR-126 mimic cells group than that in mimic-NC cells group (Fig. 7e).

### Restoring IGF1 expression improved the suppressive effects of miR-126 overexpression in cell proliferation and attenuated the promoting effects of miR-126 overexpression on cell apoptosis

**Fig. 8:**
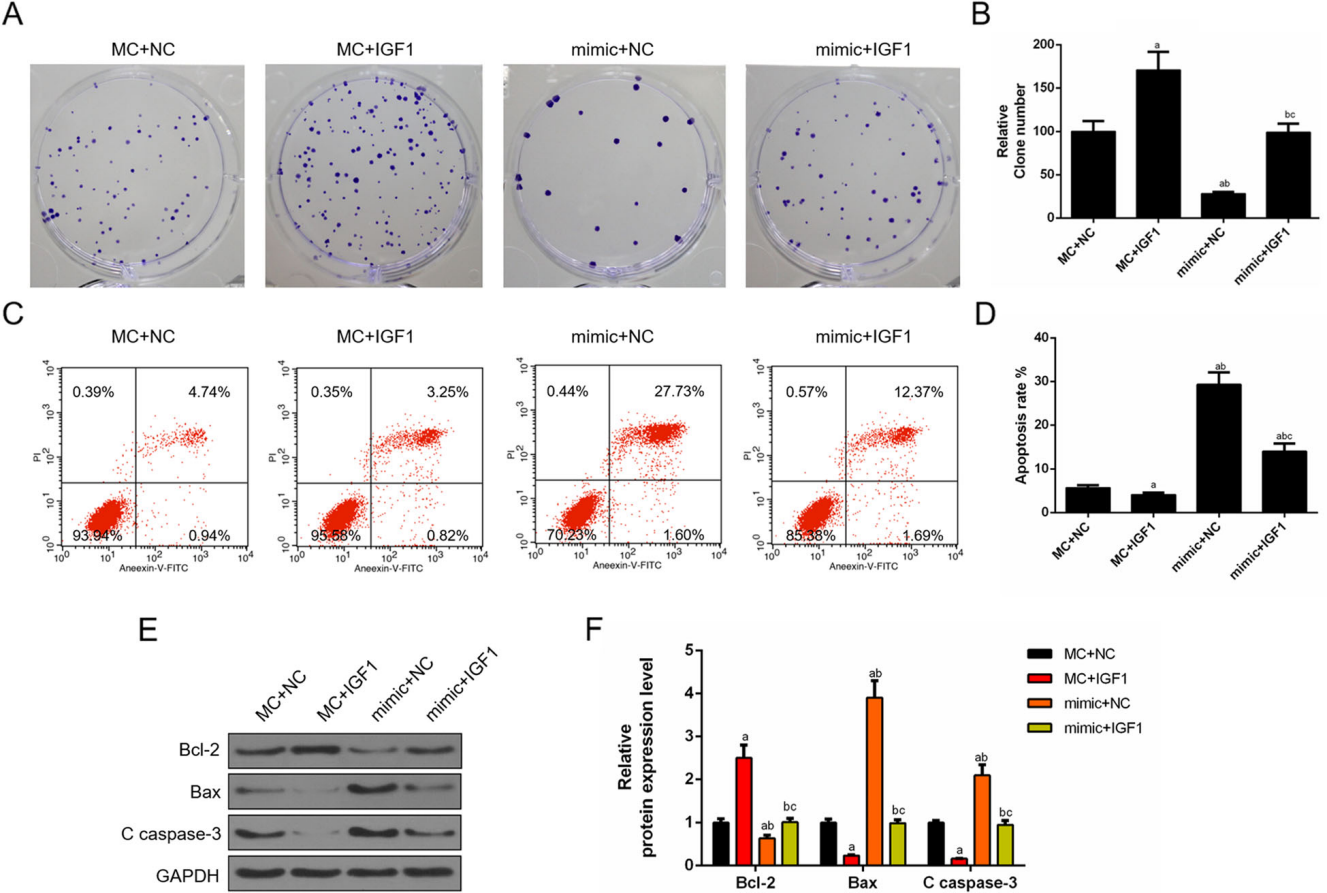
Restored IGF1 expression improved the suppressive effects of miR-126 overexpression in cell proliferation and attenuated the promotion effects of miR-126 overexpression in cell apoptosis. (**a**) Co-transfecting miR-126 mimic and IGF1 promoted cell proliferation as shown by cloning formation experiment. (**b**) The number of proliferated cells was quantified. (**c**) Cells apoptosis was triggered by miR-126 mimic, which partially reversed by IGF1. (**d**) The proportion of apoptosis was quantified. (**e**) The apoptosis-related proteins (Bcl-2, Bax and c caspase 3) were detected by Western blot. (**f**) The relative levels of proteins described in (**e**) were counted by GAPDH as normalization. ^a^*P* < 0.05 vs. MC + NC, ^b^*P* < 0.05 vs. MC + IGF1, ^c^*P* < 0.05 vs. mimic+NC

To determine whether miR-126 regulating tumor growth in vitro was correlated with IGF1, the cell proliferation and apoptosis were also determined. The results showed that the cell proliferation was enhanced by IGF1 overexpression but inhibited by miR-126 mimic, and that co-transfecting IGF1 and miR-126 mimic promoted the cell proliferation (Fig. 8a, b). The apoptosis results showed that IGF1 mitigated cell apoptosis, while miR-126 mimic obviously promoted cell apoptosis. Furthermore, cell apoptosis was triggered by miR-126 mimic, which was partially reversed by IGF1 (Fig. 8c, d). Concomitantly, the expression changes of apoptosis-related proteins Bax, Bcl2 and cleave caspase-3 were determined using Western blot, the results of which showed that IGF1 mitigated cell apoptosis, and the up-regulation of miR-126 led to the promotion of apoptosis, which was inhibited by IGF1 (Fig. 8e, f).

### The up-regulation of miR-126 suppressed the migration and invasion by regulating IGF1

**Fig. 9:**
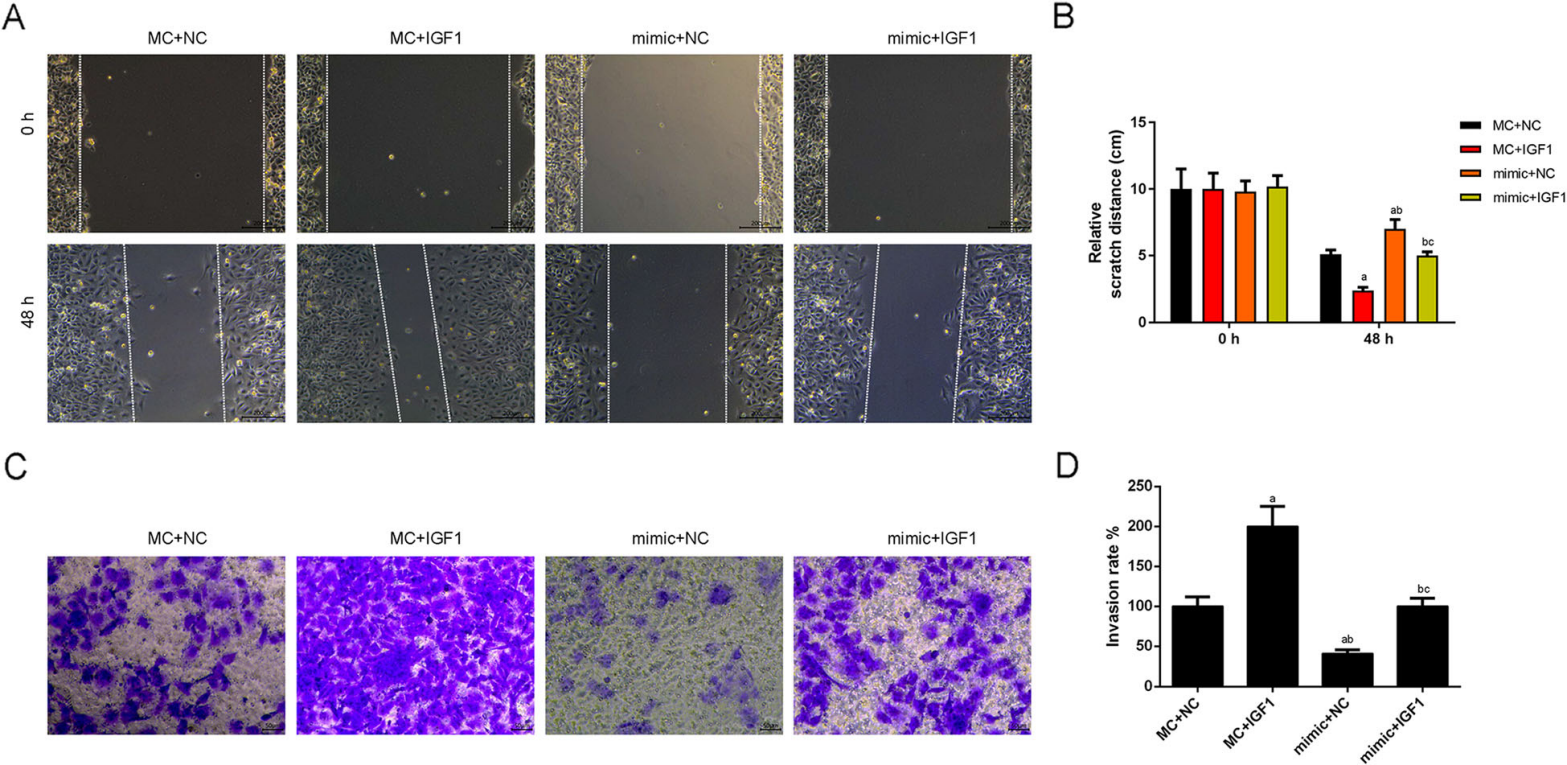
The up-regulation of miR-126 suppressed migration and invasion by regulating IGF1. (**a**) CHON-001 cells migration was inhibited by miR-126 mimic, which was reversed by IGF1 using wound healing assay. (**b**) The distance of scratch was quantified. (**c**) CHON-001 cells invasion was inhibited by miR-126 mimic, which reversed by IGF1 using transwell assay. (**d**) The numbers of invasion cells was quantified. ^a^*P* < 0.05 vs. MC + NC, ^b^*P* < 0.05 vs. MC + IGF1, ^c^*P* < 0.05 vs. mimic+NC

To further confirm that the effect of miR-126 on CHON-001 cells was correlated with the regulation of IGF1, the cell migration and invasion capacities were respectively determined. MiR-126 mimic or/and IGF1 were transfected into CHON-001 cells. Scratch test showed that the cell migration was significantly shorter in miR-126 mimic-transfected group but was higher in IGF1-transfected group than that in their control group, whereas the cell migration in miR-126 mimic+IGF1 group was significantly longer than that in miR-126 mimic-transfected group (Fig. 9a, b). Consistently, Transwell assays also produced similar experimental results (Fig. 9c, d).

## Discussion

Previous studies determined that chondrocyte survival was associated with joint destruction in OA patients [21, 22]. The human chondrocyte cell line CHON-001, derived from normal human articular cartilage, has been widely used in vitro in osteoarthritis models [23–25]. Although researches have been conducted on different aspects of OA, further studies are required to be carried out on the molecular mechanisms involved in chondrocyte survival in OA. Thus, chondrocyte-CHON-001 cells were selected for studying the molecular mechanisms underlying mimic OA injury.

Recently, increasing attentions have been focused on LncRNA, and many studies have demonstrated that lncRNAs such as SNHG5 [25], DANCR [26], MEG3 [27] and FAS-AS1 played important roles in the development and progression of OA [28]. In 2008, Watanabe T. et al found that LncRNA DNM3OS was indispensable for mouse cartilage development [17]. Recently, a study demonstrated that DNM3OS promoter containing regulatory elements is associated with chondrogenesis [18]. Combining these published studies, we hypothesized that LncRNA DNM3OS may be involved in the pathogenesis of OA, and we found for the first time that DNM3OS level was significantly lower in OA patients compared with the normal controls. Furthermore, the up-regulation of DNM3OS promoted cells viability, migration and invasion and induced apoptosis. Those data suggest that DNM3OS is a necessary factor for OA progression, at least in CHON-001 cells.

The underlying mechanism of DNM3OS in OA remains poorly understand. It is now accepted the theory that lncRNAs acted as a ceRNA for miRNAs, and in turn, miRNAs regulate their biological functions in a variety of diseases including OA [26, 29]. For example, miR-214 is highly conserved across vertebrates and is encoded within a larger non-coding RNA, DNM3OS [17]. Previous study have found that circulating miRNA expression profiles act as important roles in knee OA patients underwent celecoxib treatment, and miR-126-5p, miR-320a as well as miR-146a-5p might correlate with treatment response to celecoxib [30]. A study demonstrated that inhibition of miR-126 could protect chondrocytes from IL-1β induced inflammation via up-regulation of Bcl-2 [23]. Yang et al. have reported that Baicalin alleviated IL-1β-induced inflammatory injury via down-regulating miR-126 in chondrocytes [31]. In the present study, we discovered that miR-126 was the miRNA binding to DNM3OS. The level of miR-126 was higher in OA patient than that in normal people and was negatively correlated with DNM3OS expression. Moreover, the inhibitory effects of silencing DNM3OS on cells viability, migration and invasion were ameliorated by miR-126 inhibitor. Those findings suggest that the regulation of DNM3OS on CHON-001 cells is possibly correlated with miR-126.

A series of previous studies showed that miRNAs exerted their functional roles by regulating the expressions of endogenous targets [32]. Endogenous miR-126 suppresses metastatic endothelial recruitment through targeting of IGFBP2, which was secreted by metastatic cells recruiting endothelia by modulating IGF1 [33]. The IGF1-induced Wharton’s Jelly mesenchymal stem cells could enhance chondrogenesis [34]. In the present study, miR-126 mimics decreased the mRNA and protein expressions of IGF1. It was also found that IGF1 was involved in the regulation of miR-126 on the viability, proliferation, migration, invasion and apoptosis in CHON-001 cells. Our data indicated that DNM3OS exerted its pro-proliferative and anti-apoptotic roles via regulating miR-126/IGF1 signaling pathway in OA chondrocytes, at least in CHON-001 cells.

However, there were some limitations in our study, for example, the function of DNM3OS in protecting against CHON-001 cells injury by promoting proliferation and inducing apoptosis was only supported by in vitro experiments. Additionally, the observed significant regulation of DNM3OS/miR-126/IGF1 signaling pathway in CHON-001 cells requires further investigation.

## Conclusion

In conclusion, our study was the first to investigate the role of DNM3OS in OA chondrocytes CHON-001 cells. The results indicated that DNM3OS level was significantly down-regulated in OA tissues, and that increased expression of DNM3OS could promote the proliferation and inhibit apoptosis of CHON-001 cells. MiR-126 was a bona fide target for DNM3OS, and the biological functions of DNM3OS in CHON-001 cells were dependent on miR-126 level. We also found that IGF1 was a target gene for miR-126, and the biological functions of miR-126 in CHON-001 cells were dependent on IGF1 level. Those findings suggest that DNM3OS/miR-126/IGF1 is a signal pathway mediating the proliferation and apoptosis in CHON-001 cells, showing the potential therapeutic effects of DNM3OS in OA.

## Data Availability

The analyzed data sets generated during the study are available from the corresponding author on reasonable request.

## Abbreviations

DMEM: Dulbecco’s Modified Eagle’s Medium
DNM3OS: Dynamin 3 opposite strand
FBS: Fetal bovine serum
IGF1: Insulin-like growth factor-1
MUT: Mutated-type
OA: Osteoarthritis
PBS: Phosphate-buffered saline
PVDF: Polyvinylidene fluoride membrane
qRT: Quantitative real time
RIPA: Radio-Immunoprecipitation Assay
WT: Wild-type
